# Cardiovascular microRNAs: as modulators and diagnostic biomarkers of diabetic heart disease

**DOI:** 10.1186/1475-2840-13-44

**Published:** 2014-02-14

**Authors:** Shruti Rawal, Patrick Manning, Rajesh Katare

**Affiliations:** 1 Department of Physiology, HeartOtago, Otago School of Medical Sciences, University of Otago, Dunedin, New Zealand; 2 Department of Medicine, Dunedin Hospital, Dunedin, New Zealand

**Keywords:** Diabetic heart disease, Subclinical disease, MicroRNA, Cardiac gene expression, Diagnostic biomarkers

## Abstract

Diabetic heart disease (DHD) is the leading cause of morbidity and mortality among the people with diabetes, with approximately 80% of the deaths in diabetics are due to cardiovascular complications. Importantly, heart disease in the diabetics develop at a much earlier stage, although remaining asymptomatic till the later stage of the disease, thereby restricting its early detection and active therapeutic management. Thus, a better understanding of the modulators involved in the pathophysiology of DHD is necessary for the early diagnosis and development of novel therapeutic implications for diabetes-associated cardiovascular complications. microRNAs (miRs) have recently been evolved as key players in the various cardiovascular events through the regulation of cardiac gene expression. Besides their credible involvement in controlling the cellular processes, they are also released in to the circulation in disease states where they serve as potential diagnostic biomarkers for cardiovascular disease. However, their potential role in DHD as modulators as well as diagnostic biomarkers is largely unexplored. In this review, we describe the putative mechanisms of the selected cardiovascular miRs in relation to cardiovascular diseases and discuss their possible involvement in the pathophysiology and early diagnosis of DHD.

## Introduction

Over the past few decades, there has been a substantial rise in the population suffering from diabetes. According to International Diabetes Federation, 365 million people suffered from diabetes in 2011 and this number is expected to rise up to 552 million by 2030 [1]. Among the vast array of long-term complications associated with diabetes, cardiovascular diseases account for increased morbidity and mortality among diabetic population. Almost 80% of deaths associated with diabetes are due to heart disease [2]. Diabetic heart disease (DHD) is increasing significantly to epidemic proportions, because of the increasing obesity, sedentary lifestyle, lack of exercise and aging population. Despite all the efforts and advancement made to address this global burden, high incidence of heart disease in diabetics remains a challenge to the clinicians. Identifying the modulators of the disease will therefore not only lead to the development of novel and effective therapeutic interventions but also new ways to diagnose the disease at an early stage.

**Figure 1:**
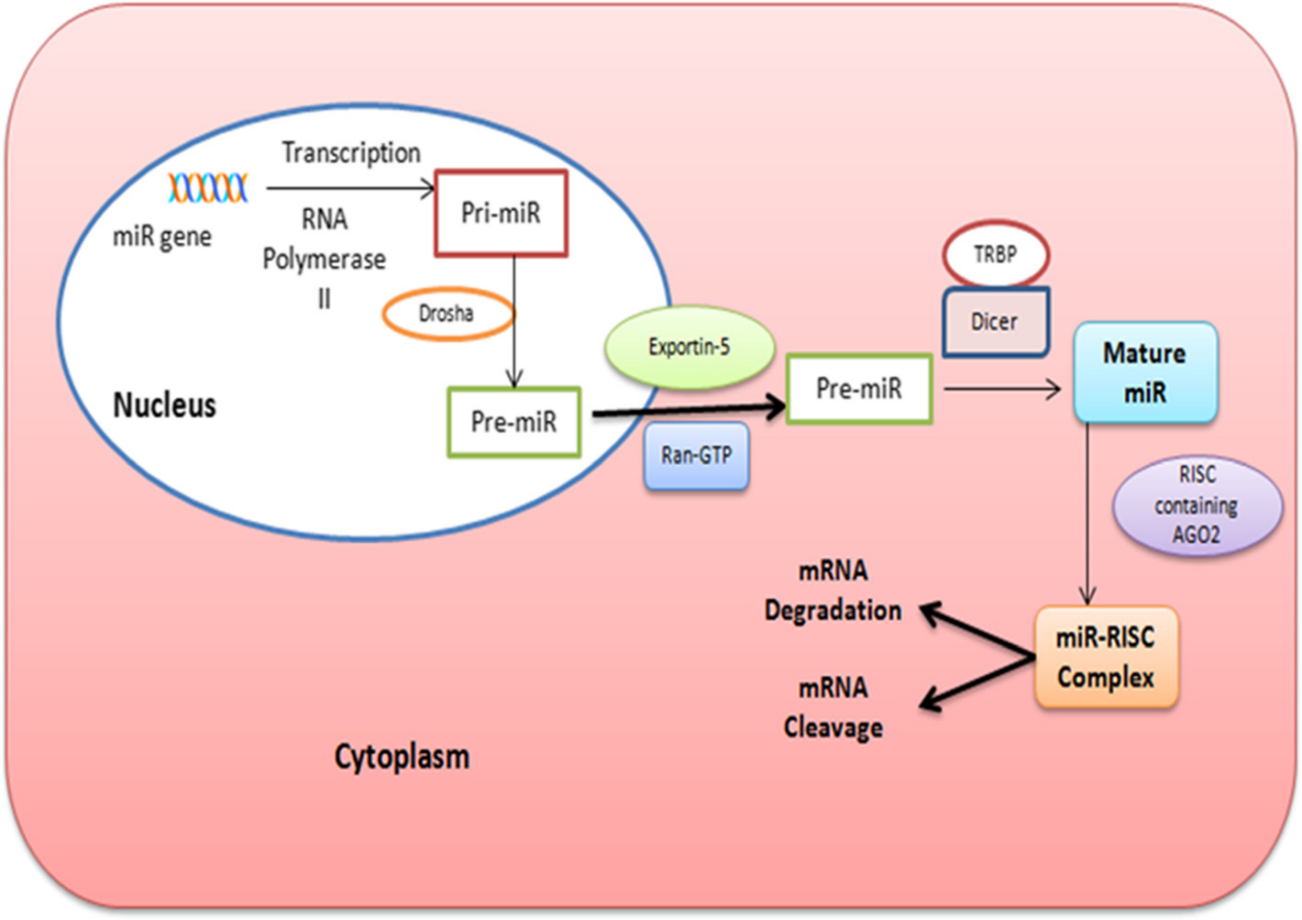
miR biogenesis and mRNA silencing mechanisms.

MicroRNAs (miRs) are a novel family of highly conserved, short (~18-25 nucleotide), non-coding single-stranded RNA molecules that regulate transcriptional and post-transcriptional gene expression [3,4]. They were first discovered as antisense RNA in the nematode *Caenorhabditis elegans* by Lee et al. [5]. Since their discovery, these small and ubiquitous RNAs have been shown to play critical roles in modulating a wide range of physiological and pathological events [6]. Biogenesis of metazoan miR is a multistep process that is initiated in the cell nucleus. Most of the miRs are located within introns of the host genes (protein-coding or non-protein-coding) or within the 3’ untranslated region (UTR) of mRNA genes. Some are found within host exons, while few others are also clustered with other miRNA. miRs that are derived from their own transcription units are located in intergenic regions of the genome [7]. miRs genes are transcribed by RNA polymerase II. Transcription of miR genes by RNA polymerase II form primary transcripts known as primary miR (pri-miR). These pri-miRs are processed by Drosha RNase III endonuclease enzyme together with its binding partner DiGeorge syndrome critical region 8 (DGCR8) that cleaves both strands of pri-miR near the base of primary stem loop to produce ~70 nucleotide long precursor miR (pre-miR).This cleavage imparts an imperfect stem-loop (hairpin loop) to pre-miR that later represents one end of the mature miR. The pre-miR is then actively transported to the cytoplasm by Ran-GTP and Exportin-5 (as shown in Figure 1). Within the cytoplasm, the hairpin loop of pre-miR is cleaved by second RNase III endonuclease enzyme, known as Dicer. Dicer, coupled with its binding partner TRBP (Transactivator RNA-binding protein) specifically recognizes the hairpin structure and cleaves the pre-miR into an siRNA-like imperfect duplex (miR: miR*) that comprises of both the mature miR strands (miR) as well as its complementary sequence on the opposing arm of pre-miR (miR*). The mature miR eventually gets incorporated into a ribonucleoprotein complex, known as RNA-induced silencing complex (RISC) to form miR-associated RNA-induced silencing complex (miR-RISC) while the miR* gets degraded. The main component of RISC is Argonaute protein which facilitates the binding of miR-RISC to the target mRNA by guiding RISC to recognize the target mRNA having complementary sequence to perform gene silencing. The mechanisms by which miRs regulate gene expression and protein translation are: 1) mRNA cleavage by binding to perfectly complementary sequence. 2) The alternative and more common mechanism is by binding to partially complementary sequence in 3’UTR of target mRNA, leading to repression of protein translation [3,8]. However, recently, miRs have also been shown to upregulate translation process during the arresting phase of cell cycle. Intriguingly, miRs oscillate between repression and activation of translation in a cell cycle; repressing translation in proliferating phase while mediating activation of translation in G1/G0 arresting phase of cell cycle [9,10], thereby fine tuning the translation of multiple target mRNAs. This could possibly explain the differential and temporal expression of miR in a variety of cardiac diseases suggesting that miRs play an essential role in regulating the pathogenesis of these diseases. A single miR can modulate complex pathological processes by acting on multiple targets (known as pleiotropic effect), thereby producing different and sometimes opposite functions depending upon the time-point of the disease. Table 1 describes the pathological role and targets of the most important cardiovascular miRs.

**Table 1:** Pathophysiological role of cardiovascular miRs in heart diseases

**S.No**	**MiR**	**Expression pattern**	**Regulators of miR**	**Phenotype**	**Reported pathophysiological roles**	**Reported/proposed role of miR in DHD**	**miR targets**	**Type of models studied**	**References**
1.	miR-1	Cardiac and skeletal muscle	SRF (specifically in heart); MEF2 and MyoD (specifically in skeletal muscles)	Cardiac & skeletal muscle proliferation & differentiation; pro-apoptotic in infarcted heart; arrhythmogenesis; inhibition of cardiac hypertrophy	Cardiac hypertrophy, apoptosis, heart failure, arrhythmia	Reported in diabetic cardiomyopathy; may also regulate diabetes-associated arrhythmia and cardiac regeneration.	Downregulation of Pim-1, calmodulin and MEF2a; Hand2, RasGAP, Cdk9, Rheb, fibronectin, HSP60 and HSP70; HDAC4, IGF-1; Cx43 protein; Kir2.1; Junctin, KCNJ2, GJA1, Irx5	TAC, transgenic mice with AKt overexpression and exercise-induced hypertrophy model in rats; neonatal rat ventricular cardiomyocytes, hypertrophic human atria and ventricles, STZ-diabetic mice, HG-exposed rat cardiomyocytes	[11-25]
2.	miR-133	Cardiac and skeletal muscle	SRF; MEF2; MyoD;	Promotes cardiogenesis; cardiac regeneration by promoting proliferation; prevents differentiation; decreases heart rate; inhibition of cardiac hypertrophy; inhibition of fibrosis in left ventricle	Cardiac hypertrophy, heart failure, arrhythmia	Reported in diabetic cardiac hypertrophy; diabetes-associated arrhythmia. May be implicated in cardiac regeneration in diabetic heart.	Downregulation of RhoA; Cdc42; Nelf-A/WHSC2; PP2A, ERG, caspase 9, SRF, KCNQ1, nPTB; CyclinD; Sp-1; CTGF	TAC, transgenic mice with AKt overexpression and exercise-induced hypertrophy model in rats; neonatal rat ventricular cardiomyocytes, hypertrophic human atria and ventricles; STZ-type I diabetic mice model; HG-exposed rat cardiomyocytes	[13,16,20,26-29]
3.	miR-126	Endothelial cells	ETS1 and ETS2	Promotes angiogenesis, anti-apoptotic	Atherosclerosis, CAD, heart failure, Myocardial infarction	May be involved in Diabetes-mediated atherosclerosis and post-MI remodeling	Upregulation of angiopoietin receptor Tie-1, c-kit, interleukin-8; CXCL12. VEGF, VEGF receptors via repression of Spred-1, Downregulation of EGFL7	k/o mice; Apoe^−/−^ mice model of atherosclerosis	[30-40]
4.	miR-208a	Cardiac muscle	αMHC	Regulation of cardiac conduction system by controlling atrial depolarization; regulates gene network in stem cell differentiation of cardiomyocytes, upregulation of βMHC	Cardiac hypertrophy, cardiac remodeling, arrhythmia; human dilated cardiomyopathy; fibrosis	May be implicated in diabetic cardiomyocyte hypertrophy, arrhythmia and in cardiac regeneration.	Downregulation of THRAP1, myostatin, βMHC	miR-208a mutant mice, k/o mice, transgenic mice with miR-208a overexpression, TAB-induced hypertrophy in mice	[41-44]
5.	miR-499	Cardiac ventricles	MRFs and Eos	Cardiac regeneration by regulation of cardiac differentiation and proliferation; cardiac gene reprogramming; anti- apoptotic; regulates stress response genes	myocardial infarction; cardiac hypertrophy; fibrosis and cardiac conduction	Diabetic cardiomyopathy. May be implicated in cardiac regeneration in diabetic heart.	Repression of histone deacetylase 4 and Sox6 proteins; Downregulation of KCNN3 gene encoding SK3 channels; Gadd45alpha; calcineurin; cyclin D1; Bmp2k, Cpne3, Hipk1, Hipk2, Map3k2 (Mekk2), Stk35, Taok1, and Uhmk1	Transgenic mice with miR-499 overexpression, TAB-induced hypertrophy, human heart, HL-1 cells transfected with miR-499 mimic, neonatal rat cardiomyocytes exposed to anoxia	[45-52]
6.	miR-132	endothelial cells, Neuron cells	CREB, VEGF	Promotes angiogenesis, regulates cardiac hypertrophy and autophagy, modulates inflammation	myocardial infarction; cardiac hypertrophy; Heart Failure; atherosclerosis and CAD	May be implicated in post-MI, heart failure and cardiac regeneration in diabetic heart.	Downregulation of p120RasGap	HUVECs, Human embryonic stem cell vasculogenesis model	[53-56]

Apart from their role in regulating the gene expression, miRs are also released in to the circulation in various packaged forms like encapsulated in microparticles (exosomes, microvesicles and apoptotic bodies) [30,57,58] or in complexes with RNA-binding proteins such as Argonaute2(Ago2) [59] or lipoprotein complexes with HDL [60]. These forms prevent the degradation of miRs in circulation and hence make them stable in circulation. Recently, miRs are gaining a lot of interest as the diagnostic biomarkers of several chronic diseases such as diabetes, cancer and cardiovascular diseases because of their high stability in the circulation and reproducibility of the results along with consistency among individuals of same species [11,61]. However, to date, they have not been investigated as diagnostic tool for DHD. This review mainly focuses on describing the pathological role of selected cardiovascular miRs that could be involved in the pathogenesis of DHD along with their salutary aspects for early detection of DHD.

### Diabetic heart disease

The term “Diabetic heart disease’ (DHD) refers to the myocardial disease that develops among people with type 1 and type 2 diabetes. DHD is a conglomeration of coronary heart disease (CHD) or coronary artery disease (CAD); Heart Failure (HF); cardiac autonomic neuropathy (CAN); and/or diabetic cardiomyopathy (DCM) [62] and is characterized by the presence of structural, molecular and functional changes.

#### Prevalence of DHD

Diabetes is an independent risk factor for heart disease and indeed, diabetics are at a greater risk of developing heart disease as compared to those without diabetes. People with diabetes tend to develop heart disease at a younger age than the age-matched non-diabetic population, which is mainly because of the presence of other co-morbidities in diabetic individuals. Importantly, the heart disease in diabetics remains largely asymptomatic which restricts its early detection [63]. Several epidemiological and clinical studies have shown a higher prevalence of heart disease among population with type 1 or type 2 diabetes [64] as compared to non-diabetic individuals [65]. The prevalence of diastolic dysfunction was found to be as high as 40-60% in individuals with type 2 diabetes [66,67]. Recently, a seven-year prospective cohort study involving patients with longstanding type 1 diabetes showed a prevalence of myocardial dysfunction in 14.5% and chronic HF in 3.7% of the patients along with an annual incidence of 0.1% and 0.02%, respectively [68]. The risk of developing HF is magnified almost twice in individuals with diabetes than those without diabetes [69,70]. In addition, the combination of diabetes and HF is also associated with increased mortality rates. Studies revealed that even after adjusting concomitant risk factors, such as hypertension, CAD, obesity, etc., diabetes remain an independent and discrete risk factor for the development of heart disease [71,72]. Evidence of increased risk of HF in diabetic population was also well documented in the Framingham study which showed a 2 fold increased risk in diabetic men and a 5 fold increased risk in diabetic women as compared to age-matched control subjects [71].

#### Molecular alterations in DHD

DHD is often unrecognized in the early or subclinical stage due to the absence of pathognomonic signs. The early stage of DHD is usually asymptomatic and all the myocardial changes start at the molecular level. At this stage, there is insignificant alteration in myocardial structure and function that could be clinically assessed. Diabetes-associated metabolic anomalies such as hyperglycemia, hyperlipidemia, inflammation and insulin resistance initiate a series of molecular events in the myocardium. These molecular events are characterized as increased circulating free fatty acids (FFAs); enhanced oxidative stress, advanced glycation end products (AGE); endothelial dysfunction; activation of protein kinase C (PKC), poly (ADP-ribose) polymerase (PARP) and polyol pathway along with altered calcium homeostasis [73,74]. These anomalies also cause dysregulation of various molecular pathways leading to accelerated apoptosis, reduced reparative angiogenesis, impaired electrical conduction and maladaptive remodeling.

Changes in cellular signaling pathways are the early defects that have been described extensively in several animal models. A recent study by Erickson et al. [64] showed alteration in myocadial Ca^2+^ ion channels, Ca^2+^ homeostatic proteins, and transcription factors due to hyperglycemia-mediated activation of Ca^2+^/calmodulin-dependent protein kinase II (CaMKII) which consequently aggravate arrhythmia in diabetes. Intriguingly, a study by Chen et al. [75] investigated hyperglycemia-induced alteration in expression of cardiac ATP sensitive potassium (K(ATP)) channels during the early stages of diabetes, that might account for metabolic alterations and cardiac dysfunction in diabetes. A reduced gene expression of (K(ATP)) channels was also observed in heart of streptozotocin (STZ)-induced diabetic rats after 8 weeks of induction. Interestingly, the decrease in gene expression was restored by insulin treatment, epitomizing the need for glycaemic control of diabetes at early stage to lower the associated complications. We have previously reported a blunted activation of VEGFR-2/Akt/Pim-1 pro-survival signaling pathway in STZ-induced type 1 diabetic mice which consequently resulted in increased cardiomyocyte apoptosis, reduced cardiac contractility and neovascularization [76]. Further, we observed the change in expression and activity of STAT3/Akt/Pim-1 signaling in the early stage of DCM. Of note, these alterations started appearing in the initial stage of the disease before the manifestation of overt failing heart, reflecting the onset of the disease much earlier at molecular level [77]. Intriguingly, systemic administration of Pim-1 in STZ-induced type 1 diabetic mice via cardiotropic adeno-associated virus serotype-9 vector [12] or administration of benfotiamine, a vitamin B1 analog at the initial stage [77] halted the progression of the disease. Both these studies suggest that early detection and timely management can prevent the progression of heart disease in diabetic individuals.

Diabetes-mediated molecular alterations activate a web of interconnected stress signaling pathways within the myocardium that culminate with the activation of various transcription factors, co-regulators and miRs, leading to alterations in gene expression in diabetic heart [78,79]. Several studies have demonstrated that aberrant cardiac gene expression contributes to structural and functional changes which are manifested as hypertrophy, altered cardiac conduction, reduced contractility and cardiomyocyte survival along with disturbances in vascular homeostasis [80-82]. Using Otsuka Long-Evans Tokushima Fatty (OLETF) rat model of type 2 diabetes, Karakikes et al. [81] investigated the changes in cardiac gene expression and the subsequent alteration in intracellular signaling pathways in DCM. DCM was found to be associated with hypertrophy; dysregulation of insulin signaling pathway and glucose metabolism; and contractile dysfunction associated with abnormal calcium handling along with downregulation of sarcoplasmic reticulum Ca^2+^ ATPase (SERCA2) protein expression. Microarray analysis revealed a total of 838 genes to be differentially expressed between control and diabetic hearts, of which, 272 genes were upregulated and 566 genes were downregulated, indicating the aberrant cardiac gene expression in diabetes. Furthermore, adenoviral transduction of SERCA2a gene in 60–65 week old OLETF rats resulted in reversal of cardiac hypertrophy, fibrosis, ventricular arrhythmias as well as contractile dysfunction, indicating the improvement of myocardial performance post-SERCA2a gene transfer. In addition, a set of 76 genes were found to be modulated with SERCA2a gene transfer. Of particular interest, the expression profile of insulin-signaling molecules (PI3K, Akt, Glut4 and PKCλ) was restored after SERCA2a gene transfer. Similarly, overexpression of SERCA2a in isolated rat cardiomyocytes modulated the expression profile of major transcripts involved in insulin signaling, glucose metabolism and cardiac remodeling. Similarly, a recent study by Sárközy et al. [82] on male Zucker Diabetic Fatty (ZDF) rats, a model of metabolic syndrome, showed altered gene expression pattern in diabetic myocardium, with 36 genes being upregulated and 49 genes being downregulated as compared to the normal hearts. Collectively, these findings indicate marked alteration in cardiac gene program in diabetic heart and therefore, augment the need to explore the modulators of gene expression in diabetic heart.

In a study by Feng et al., glucose-induced hypertrophy of neonatal rat cardiomyocytes was accompanied by upregulation of a transcriptional coactivator, p300 and activation of p300-dependent Myocyte Enhancer Factor-2(MEF-2): MEF2A and MEF2C. MEF2 transcription factors control the expression of many fetal cardiac genes and are reactivated in cardiac hypertrophy [83]. Intriguingly, blockade of p300 using curcumin and p300 siRNA (small interfering RNA) inhibited the interaction of p300 with transcription factors, thus preventing the upregulation of the transcription factors involved in cardiac hypertrophy in STZ-diabetic rats. Much of our current understanding on the regulatory phenomenon of cardiac gene expression is at transcriptional level, where transcriptional factors are known to govern the activation of specific genes. However, recent studies have uncovered another layer of cardiac gene regulation, with a single gene being controlled by multiple regulators at post-transcriptional level. Indeed, the transcription factors have been found to drive the cardiac processes via regulating the gene expression of specific miRs, which act as post-transcriptional modulators of cardiac gene expression [13,84].

### Functional role of miRs in DHD

**Figure 2:**
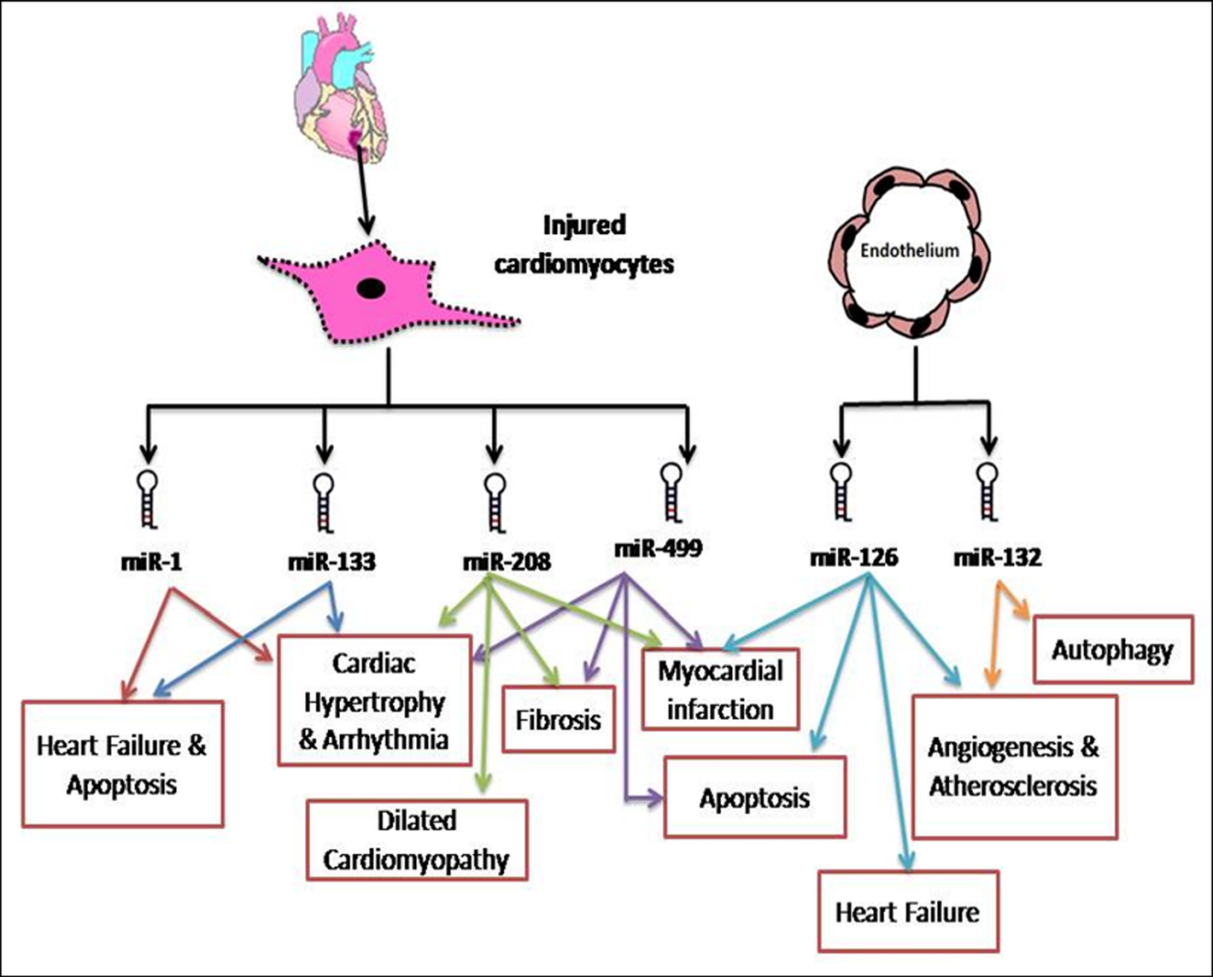
Pathological roles of cardiovascular miRs in heart diseases.

miRs have been defined as the “micromanagers” of gene expression [85]. The discovery of miRs as the regulators of multiple cardiac genes has added a new mechanistic link between gene regulation in the normal and failing heart at post-transcriptional level. They might act as fine-tuners of the expression of mRNAs, by on-off switching mechanism. Recently, miRs have been identified as a key element involved in cardiac gene remodeling in diabetic heart [78]. A host of recent studies suggests that miRs play a crucial role in the pathogenesis of diabetes and many cardiovascular complications such as endothelial dysfunction, angiogenesis, hypertrophy, arrhythmia, HF and myocardial fibrosis [31,86-89] by regulating the expression of multiple genes, as shown in Figure 2 and Table 1. As these processes involve the dysregulation of multiple genes, it is reasonable to hypothesize that miRs could be implicated in the pathogenesis of DHD. An insight in to the molecular mechanisms and targets of miR involved in the pathogenesis of heart diseases could serve as a prerequisite to explore their role in the pathogenesis of DHD. Additionally, molecular markers that could track the aberrant gene expression under pathological conditions could serve as useful diagnostic markers for early detection of disease at molecular level. For a comprehensive understanding of the pathological and diagnostic potential of miRs in DHD, it is important to understand the identified miR targets under various cardiovascular diseases.

### Pathological role of miR in cardiovascular disease

A subset of miRs that are either specifically or highly expressed in cardiac and vascular cells are termed as cardiovascular miRs, such as miR-133, miR-1, miR-208, miR-499, miR-126 and miR-132. Considering the largely undetermined role of these miRs in DHD, we decided to focus on describing the possible involvement of this subset of miRs in DHD, with reference to their established role in various cardiovascular diseases. A detailed pathological role of each of these miRs in cardiovascular disease has been described in this section.

#### miR-133 and miR-1

miR-133 and miR-1 are reported to be expressed specifically in cardiac and skeletal muscles [90]. miR-133 family comprises of 3 members: miR-133a-1(expressed in cardiac and skeletal muscles), miR-133a-2(expressed in cardiac and skeletal muscles) and miR-133b (expressed specifically in cardiac muscles); each of which share a common bicistronic unit with miR-1 and miR-206. However, all these miRs are expressed as separate transcripts and exhibit different phenotypes [14]. miRs that belong to miR-1 family are designated as miR-1-1 and miR-1-2 and are expressed in both cardiac and skeletal muscles. [14]. Although clustered on the same chromosomal loci, miR-133 and miR-1 are reported to play distinct roles in modulating cellular processes in myoblasts [84,91]. miR-133 was found to enhance myoblast proliferation by repressing serum response factor (SRF) whereas miR-1 was found to induce myogenic differentiation by targeting histone deacetylase 4 (HDAC4), a transcriptional repressor of muscle gene expression. miR-1 has also been reported to control muscle differentiation by targeting Hand2, a transcription factor that regulates expansion of ventricular cardiomyocytes [15]. Intriguingly, the expression of miR-1/miR-133 cluster is regulated by multiple transcriptional factors: SRF [15]; MEF2 [13] and MyoD [16]. Several studies have demonstrated the important role of miR-1 and miR-133 in various cardiovascular diseases. However, their role in DHD is still unrecognized.

##### Cardiac hypertrophy

Carè and colleagues were the first to demonstrate the critical role of miR-133 in cardiac hypertrophy [88]. Their study described the inverse correlation of miR-133 expression and cardiac hypertrophy in mouse and human models of hypertrophy. The report also indicated the specific targets of miR-133 as: RhoA, a GDP-GTP exchange protein regulating cardiac hypertrophy; Cdc42, a signal transduction kinase implicated in hypertrophy; and Nelf-A/WHSC2, a nuclear factor which is involved in cardiogenesis. Impairment in cardiac glucose transport system has also been implicated in the pathogenesis of failing and hypertrophic hearts [92]. Translocation of GLUT4 to the plasma membrane is the major mechanism of glucose uptake in cardiomyocytes to maintain myocardial energetics during ischemic stress [93]. This translocation is triggered by some responsive factors like insulin, ischemia and exercise. miR-133 overexpression was found to reduce the GLUT4 glucose transporter levels in cardiomyocytes along with reduced insulin-induced glucose uptake by repressing the protein KLF15 [94] suggesting that miR-133 may be suppressing the hypertrophy through regulation of the cardiac glucose transport system. In support of the above finding, another study using STZ induced type 1 diabetes model showed marked decrease in the level of miR-133a which was associated with hypertrophy and contractile dysfunction at 2 months after induction of diabetes [95]. Furthermore, in vitro exposure of rat neonatal cardiomyocytes to high glucose (HG, 25 mmol/L) for 48 h produced hypertrophic changes in cardiomyocytes along with reduced expression of miR-133a. This was also in conjunction with increased protein expression of the transcriptional regulators of miR-133, MEF2A and MEF2C. Finally, the investigators transfected the HG-exposed cardiomyocytes with custom-synthesized miR-133a mimics and observed the attenuation of hypertrophy along with downregulation of SGK1 and IGF1R mRNAs, the targets of miR-133a in cardiac hypertrophy. The study elucidated the effect of miR-133a on HG-cardiac hypertrophy by downregulating SGK1 and IGF1R mRNAs. In lines of the above mentioned evidences, it can be suggested that miR-133 play a pathophysiological role in regulating the hyperglycaemia-induced hypertrophy in diabetic human heart. However, further studies are still required to investigate the potential role of miR-133 in regulating cardiac hypertrophic changes in human failing hearts associated with diabetes. In addition to its effects on cardiomyocytes, miR-133 is also documented as a key regulator of vascular smooth muscle cell phenotypic modulation in vitro and in vivo by regulating the expression of SP-1, suggesting its potential therapeutic application for vascular diseases [26].

Sayed et al. [17] demonstrated the downregulation of miR-1 as early as day 1 after transverse aortic arch constriction (TAC)-induced hypertrophy in mice. Later on, gain and loss-of-function studies done by Carè et al. [88] showed significant decrease in the expression of miR-1 in three different murine models of hypertrophy: TAC mice, transgenic mice with AKt overexpression and exercise-induced hypertrophy model in rats. In line with this, adenoviral-mediated overexpression of miR-1 attenuated endothelin-1 induced hypertrophy in neonatal rat ventricular cardiomyocytes along with growth-related targets such as Ras GTPase-activating protein (RasGAP), cyclin-dependent kinase 9 (Cdk9), fibronectin and Ras homolog enriched in brain (Rheb). Ikeda et al. demonstrated that miR-1 mediated regulation of hypertrophy is through downregulation of the calcium-calmodulin signaling [18]. Recent studies have revealed the critical role of miR-1 in regulating cardiac injury by repressing the expression of cytoprotective signaling proteins such as heat shock proteins (HSPs), insulin growth factor 1(IGF-1), protein kinase C epsilon (PKCϵ) and BCL2 [96-98]. PKCϵ is a member of protein kinase C (PKC) family that is known to play protective roles in cardiac ischemia-reperfusion injury [99]. HSP60 is an anti-apoptotic protein in mitochondria that inhibit cell apoptosis by forming complexes with apoptotic proteins like Bax, Bak and Bcl-xS [100]. Shan et al. [97] reported the increased levels of miR-1 and miR-206 in HG-treated rat neonatal cardiomyocytes and rat myocardium through modulation of SRF and MEK1/2 signaling pathway. In addition, miR-1/miR-206 was found to repress HSP60, thereby contributing to HG-induced apoptosis in rat cardiomyocytes. HSP60 is recently been discovered as an important component of the defence system against diabetic myocardial injury and its level is reduced in the diabetic myocardium through upregulation of miR-1 [97,101]. These studies provide clear evidence for the critical role of miR-1 in the pathophysiology of the DHD. In our previous study [12], we reported an upregulation of cardiac miR-1 and the resultant decrease in one of its target protein Pim-1 as a causative factor for the development of cardiomyopathy in STZ-induced type 1 diabetic mice. However, forced expression of Pim-1 using an adeno-associated virus serotype-9 halted the progression of DCM by activation of the prosurvival pathway and conservation of sarcoendoplasmic reticulum Ca^2+^-ATPase. In contrast, a very recent study by Yildirim et al. [19] showed downregulation of miR-1, miR-499, miR-133a, and miR-133b and upregulation of miR-21 in STZ-induced diabetic rats. The authors suggested an inverse relationship of these downregulated miRs with increased oxidative stress in diabetic myocardium. Intervention with N-acetylcysteine (NAC), an antioxidant for 4 weeks starting from immediately after diabetes induction resulted in normalization of cardiac function along with restoration of expression levels of miR-499, miR-1, miR-133a, and miR-133b in the ventricular cardiomyocytes. It should be noted that controversies exist among these two independent studies, which could be due to the different time point where the levels of miR-1 was measured as well as due to difference in the species used between each study. These contrary reports therefore, invite a further extensive investigation into the pathophysiological role of miR-1 at different stages of DHD.

##### Arrhythmia

As a consequence of ischemia, hypertrophy and necrosis, cardiac cells undergo electrical reorganization which impairs the normal conduction system within the heart, thereby increasing the risk of developing arrhythmia. Management of arrhythmia requires the better understanding about the therapeutic targets. Studies revealed that people with diabetes are at a much higher risk of developing atrial fibrillation (AF) and the risk is more in individuals with history of longstanding diabetes and uncontrolled blood glucose levels [102]. A host of recent studies indicates the modulatory role of cardiac-specific miRs in cardiac electric disorders. Overexpression of miR-1 and miR-133 are reported to increase arrhythmogenesis in canine model of non-ischemic HF due to enhanced phosphorylation of ryanodine receptor (RyR2) by depressing the activity of phosphatase, protein phosphatase 2A (PP2A) [20]. Yang et al. [21] demonstrated the overexpression of miR-1 in coronary artery disease (CAD) patients providing a causal link between miR-1 and arrhythmia. Further, injection of miR-1 in to the non-infarcted rat heart as well as infarcted rat heart exacerbated arrhythmogenesis. For this, rats were subjected to myocardial infarction for 12 h which corresponds to the peri-infarction period in CAD patients, and similar to the clinical scenario the infarcted rat hearts showed high incidence of premature ventricular beats, tachycardia and ventricular fibrillation. Interestingly, arrhythmic effects were also observed in normal heart injected with miR-1. Importantly, these effects were rescued by administration of miR-1 specific antisense oligoribonucleotides, demonstrating the arrhythmogenic potential of miR-1. Post-transcriptional repression of 2 major ion channel genes: KCNJ2 and GJA1 and downregulation of gap junctional protein connexin-43 have been demonstrated to be the mechanism for miR-1 mediated arrhythmias [30,65].

Recent studies have shown that miRs regulate various physiological processes in a dosage-dependent fashion and variability occurs even among the members of same family. Disruption of just one of the two members of miR-1 family, miR-1-2 causes profound consequences in the development and maintenance of cardiac physiology. Zhao et al. [22] reported dysregulation of cardiogenesis and widening of QRS complex in miR-1-2 knockout mice along with abnormal cell cycle, demonstrating the crucial role of miR-1-2 in cardiac development, electrical conduction and cell cycle regulation. Using loss-of-function approach, the investigators determined the potential target of miR-1-2 as Irx5. Irx5 is a cardiac transcription factor that functions with another repressor gene, Smyd1 to repress the expression of potassium channel, Kcnd2 in ventricular myocytes. A dosage-dependent regulation of Kcnd2 by transcription factor, Irx5 is required for normal ventricular repolarization and cardiac rhythm. miR-1-2 knockout leads to shortening of PR interval and prolonged QRS complex due to increased activity of Irx5 which clearly demonstrated a disruption in cardiac rhythm and predisposition to arrhythmias. Results of these studies indicate that members of the same family of miRs may work independently to execute normal physiologic functions and also explains that dose-sensitivity of miR-regulated proteins is involved in development and maintenance of normal cardiac physiology. Electrical remodeling in DCM has been recently ascribed to reduced expression of potassium channels in myocardium and is characterized by decreased repolarization reserve and disruption in ion channels [103]. It is credible to mention here that understanding the molecular targets of miR-1 involved in the pathogenesis of arrhythmia could also serve as potential therapeutic targets for diabetes-mediated arrhythmia.

miR-133 has also been reported to play a pathological role in diabetes-associated arrhythmia [27]. Overexpression of miR-133 in diabetic rabbit hearts was shown to repress ether-a-go-go related gene (ERG) that is known to encode for K^+^ channel (I(Kr)) in cardiomyocytes. Repression of ERG by miR-133 causes depression of I(Kr) and contributes to slow repolarization, thereby producing QT prolongation and the associated arrhythmias, in diabetic hearts [101].

#### miR-208

miR-208a is a cardiac-specific miR that is encoded within an intron 29 of Myh6 gene encoding alpha myosin heavy chain (αMHC) and is regulated by αMHC. It is a member of miR-208 family that also includes miR-208b which is encoded within an intron 31 of Myh7 gene encoding beta myosin heavy chain (βMHC) and expressed in cardiac and skeletal muscles. The third member miR-499 is encoded by intron 19 of Myh7 together with miR-1, miR-133a/b, miR-208a/b and are recently determined to be important regulators of genetic network in the differentiation of stem cells in to cardiomyocytes [41].

##### Cardiac hypertrophy and arrhythmia

The contractile efficiency of myocardium is largely dependent on the contractile protein, MHC. miR-208 is reported to regulate the expression of βMHC and plays a pathological role in cardiac hypertrophy and consequently arrhythmia. After birth, the thyroid hormone (T3) increases the expression of miR-208a while repressing the expression of βMHC under normal conditions. However, under stress conditions, the fetal gene system in heart is triggered and increases the expression of βMHC while decreasing the expression of αMHC without altering the expression of miR-208a. Indeed, miR-208a functions extravagantly to regulate cardiac contractility in response to stress such as hypertrophic growth accompanied by fibrosis and thyroid hormone signaling. A study by van Rooij et al. [42] demonstrated that miR-208a, encoded by intron of αMHC is required for cardiomyocyte hypertrophy, fibrosis, and increased expression of βMHC in response to stress and hypothyroidism in adult mice. After thoracic aortic-banding (TAB)-induced hypertrophy, wild-type (WT) mice showed pronounced increase in hypertrophic growth or fibrosis. In contrast, miR-208a mutant mice showed blunted hypertrophic growth, fibrosis and a reduction in cardiac contractility in response to TAB. Importantly, the mutant mice were unable to upregulate βMHC expression in heart. Further, T3 signaling was blocked in WT and mutant mice by feeding PTU-containing chow for 2 weeks. Northern blot analyses in WT hearts showed a switch of αMHC to βMHC in response to PTU whereas the mutant amice hearts were resistant to upregulation of βMHC. Overall, the study revealed that miR-208 is necessary for upregulation of βMHC and cellular remodeling in heart through a mechanism involving thyroid hormone receptor (TR). In lines of these evidences, Callis et al. [43] reported cardiac hypertrophy and arrhythmia in mice with transgenic overexpression of miR-208a, which consequently repressed the expression of two negative regulatory target proteins: Thyroid hormone receptor-associated protein 1 (THRAP1) and myostatin. miR-208a knockout mice showed reduced hypertrophy, suggesting the crucial role for miR-208a in hypertrophic growth and cardiac contractility. In contrast, long-term genetic deletion of miR-208a resulted in decreased cardiac contractility which may possibly be due to altered cardiac conduction system causing AF [42]. This may be attributed to altered expression of cardiac transcription factors: homeodomain-only protein, GATA4 and the gap junction protein connexin 40 [43]. The above two studies indicate that partial blockade of miR-208a can halt the hypertrophic growth whereas complete knockout may initiate arrhythmia under stress conditions. As described above it is now well established that cardiac hypertrophy is the major contributor of the pathogenesis in the DCM and HF. Increased expression of βMHC has been observed in the heart of diabetic rat [104,105]. Although there is no direct study on miR-208 in context of DHD, the general role of miR-208 in regulating cardiac hypertrophic growth through MHC could suggest its pathological role in DHD.

#### miR-499

##### Cardiac hypertrophy

miR-499 is one of the three members of myomiR family that regulate the expression of myosin isoform in cardiac hypertrophy [106]. It is an evolutionarily conserved muscle-specific miR embedded within a ventricular-specific myosin heavy chain gene, Myh7b and is highly expressed in normal heart. However, its expression is modulated in cardiac stress and apoptosis [45]. Shieh et al. [46] investigated the expression pattern of miR-499 in human heart tissue and transgenic mice. Microarray analysis done on human fetal heart and liver tissues confirmed that miR-499 is enriched in human heart. In experiments using transgenic mice, the elevated levels of miR-499 resulted in cardiac hypertrophy and cardiac dysfunction in a dose-dependent manner, which was in consistent with another study [107] also showing the elevated levels of miR-499 in human HF. Further, thoracic aortic banding (TAB)-induced hypertrophy in TG mice overexpressed with miR-499 demonstrated that these mice were predisposed to increased cardiac dysfunction upon cardiac pressure overload compared to banded wild-type controls. To investigate the mechanistic targets of miR-499 involved in pathogenesis of stress-induced cardiac hypertrophy, Shieh et al. assessed the global gene expression profiles, and observed altered levels of the immediate early stress response genes (Egr1, Egr2 and Fos), ss-myosin heavy chain (Myh7), and skeletal muscle actin (Acta1). Further, gain- and loss-of- function studies in vitro confirmed the effect of miR-499 on the immediate early gene response to cardiac stress. These findings suggest the crucial role of miR-499 in regulating cardiac response to stress through regulation of stress response genes. Knowing the regulatory potential of miR-499 on cardiac stress response genes, it would be interesting to find out the response of miR-499 under diabetes induced cardiac stress.

##### Arrhythmia

Recently, miR-499 has been investigated as an important regulator of gene expression by regulating the genes involved in electrical remodeling in AF [47]. miR-499 was found to repress the expression of a small-conductance calcium-activated potassium channel 3 (SK3 channel) which is associated with AF [108]. This channel is encoded by KCNN3 gene [109]. Transfection of miR-499 mimic in HL-1 cells caused downregulation of SK3 protein expression, while that of miR-499 inhibitor resulted in upregulation of SK3 expression. This effect may be attributed to the altered expression of KCNN3 gene by miR-499. These findings suggest a molecular regulatory mechanism of miR-499 in electrical remodeling in patients with AF. Having demonstrated the role of miR-499 in the pathogenesis of arrhythmia, it is plausible that it could be also regulate the molecular targets in diabetes-associated arrhythmia.

##### Cardiomyopathy and acute myocardial infarction

miR-499 has been shown to be downregulated under pathological conditions such as ischaemia and cardiac remodeling. It has been found to regulate the proliferation and apoptosis in late stage of cardiac differentiation via its effects on Sox6 and cyclin D1 [45]. In another study, transgenic overexpression of miR-499 has been reported to blunt cardiac apoptosis and infarction via suppression of calcineurin-mediated dephosphorylation of dynamin-related protein-1 (Drp1) [48]. Drp1 is involved in initiation of apoptosis via dysregulation of mitochondrial fission machinery, which lead to cell death in myocardial infarction [110-112]. Further, antagomir-mediated knockdown of miR-499 aggravated cardiac remodeling as manifested by hypertrophy and impaired cardiac functions. In contrast, another group of studies showed cardiac dysfunction in miR-499 Tg mice through regulation of early stress response genes [46]. To validate these conflicting reports on variable expression of miR-499, Matkovich et al. [49] studied the associations between regulatory effect of miR-499 in clinical and experimental cardiomyopathy. The study revealed that upregulation of miR-499 contributes to pathological aspects of human and murine HF through direct and indirect regulation of myocardial mRNAs and proteins; modulation of cardiac kinase and phosphatase pathways along with post-translational modification of myocardial proteins. Importantly, the direct and indirect regulation of kinases by miR-499 alters the phosphorylation of key proteins which consequently influence the mitogen-activated protein kinase (MAPK) pathway, thereby controlling mRNA translation and apoptosis. These findings could explain the anti-apoptotic mechanism of miR-499 shown by Wang et al. [48], where suppression of apoptosis by miR-499 can be attributed to its post-translational regulatory effect on phosphatase, calcineurin and Drp1 proteins.

Wang et al. [50] documented the negative regulatory effect of miR-499 on Gadd45α gene expression in STZ-induced diabetic heart and diabetic dorsal root ganglia (DDRG). Gadd45α gene has been shown to directly regulate the expression of 11 different proteins that are involved in the pathophysiology of diabetes mellitus. The study also suggested a potential link of Gadd45α with DCM and baroreflex dysfunction. Expression profiling in left ventricle tissue from 4-week STZ-induced diabetic rats revealed differential expression of miR-499 and Gadd45α gene. Expression of miR-499 and Gadd45α were significantly increased at mRNA level whereas the expression of Gadd45α was robustly decreased at protein level in both diabetic heart and nucleus ambiguous, indicating a negative regulation of Gadd45α gene by miR-499. Results of this study therefore suggest a key role for miR-499 in the pathophysiology of DCM. In support to this, a recent study by Chavali et al. [113] revealed the downregulation of miR-499 in the diabetic heart, suggesting miR-499 as a putative candidate involved in the pathophysiology of DHD.

#### miR-126

miR-126, also known as “*angiomir*” is expressed only in endothelial cells and is encoded by intron 7 of epidermal growth factor-like domain 7 (EGFL7) [32]. miR-126 has been reported to regulate myocyte apoptosis [30,33], post-MI cardiac regeneration and vascular inflammation [34]. This endothelium-enriched miR is reported as a key regulator in the pathophysiology of atherosclerosis, myocardial infarction and HF. Studies revealed that miR-126 gets regulated in the reperfused heart to insulate the myocardium from perturbations due to ischemia. Indeed, it produces cardioprotective effect by repressing the post-infarct remodeling.

##### Angiogenesis

The vasculoprotective role of miR-126 has been well documented by several studies [30,35,114-116]. miR-126 has been reported to maintain the vascular integrity and angiogenesis by enhancing proangiogenic properties of vascular endothelial growth factor (VEGF) and fibroblast growth factor (FGF) and promotes neovascularization by repressing the expression of Spred-1, an intracellular inhibitor of VEGF [36,117]. This was evident in mice with knockdown of miR-126, where profound vascular abnormalities and reduced survival were observed following experimental MI. While 70% of wild type mice survived under similar surgical conditions, none of the miR-126 null mice survived beyond 3 weeks post-surgery. Consistent with these findings, antagomir-mediated silencing of miR-126 in wild type mice also showed impaired angiogenesis [37], confirming the crucial role of miR-126 in maintenance of vascular integrity and promoting angiogenesis post myocardial infarction. Zernecke et al. [30] demonstrated the atheroprotective effect of miR-126 by inducing the expression of chemokine, CXCL12, which acts as a signal to mobilize progenitor cells and as an anti-apoptotic factor. The authors showed that the delivery of miR-126 encapsulated in apoptotic bodies, to the site of atherosclerotic plaques in apolipoprotein E-deficient (Apoe^−/−^) mice resulted in knockdown of the regulator of G-protein signaling 16 (RGS16) and enable CXCR4, a G-protein coupled receptor to trigger the phosphorylation of ERK1/2, which induces the production of CXCL12. This paracrine vasculoprotection imparted by miR-126 confers its regulatory role in atherosclerosis and demands future investigations to assess its potential in diabetic subjects with coronary artery disorder. Of note, diabetes is one of the major risk factor for the development of atherosclerosis.

##### Heart failure

Fukushima et al. [118] demonstrated reduced circulating levels of miR-126 in patients with ischaemic heart disease. Interestingly, the expression level of miR-126 was negatively correlated with the age, New York Heart Association (NYHA) class and brain natriuretic peptide expression, indicating its potential as a biomarker for HF. In line with this Zampetaki et al. demonstrated low expression levels of miR-126 in plasma samples of type 2 diabetic patients [31], suggesting it as an emerging diagnostic tool for HF in patients with type 2 DM. Interestingly, miR-126 was also identified to be downregulated in atherosclerotic coronary artery disease [119], one of the major cardiovascular complication in diabetic individuals.

#### miR-132

Although not specifically expressed in heart, miR-132 has been recently found to play a pivotal role in cardiac pathophysiology. miR-132 is encoded on human chromosome 17 and is transcribed by a transcription factor cAMP-response element binding protein (CREB) in different cell types [53].

##### Angiogenesis

Anand et al. [54] demonstrated the proangiogenic property of miR-132 through downregulation of the anti-angiogenic protein p120RasGap in human umbilical vein endothelial cells (HUVECs). In addition, miR-132 was found to be upregulated in human embryonic stem cell model of vasculogenesis (a cell culture model in which embryoid bodies derived from human embryonic stem cells form well defined endothelial networks after 14 days in culture) and in the endothelium of human tumors and hemangiomas but was undetectable in normal endothelium. Further, intraocular injection of miR-132 antagomir (10 μg) induced postnatal retinal vascular development in mice, suggesting the vasculoprotective role of miR-132. The investigators also concluded that the phosphorylation of CREB by angiogenic growth factors such as VEGF, bFGF and conditioned media from different types of tumors causes activation of miR-132 levels. The loss-of-function studies confirmed the effect of miR-132 on endothelial proliferation, tube formation in vitro and both developmental and pathological angiogenesis in vivo via downstream of multiple growth factors involved in regulating endothelial proliferation, migration, and vascular patterning [120]. We had recently demonstrated the important role of miR-132 in mediating the stem cells induced post-infarct revascularization [55]. Saphenous vein progenitor cells (SVPs) markedly released miR-132 under stress conditions and when transplanted to the mice hearts subjected to myocardial infarction they activated neovascularization through the release of miR-132 in to the surrounding region. Importantly inhibition of miR-132 in the SVPs markedly retarded the therapeutic effect of stem cells thus confirming the importance of miR-132 in post-ischemic repair. Based on these evidences, it is plausible that the angiogenic and neovascularization effect of miR132 overexpression could be beneficial in post-ischaemic vascular reparative process in diabetic HF.

##### Cardiac hypertrophy

Conversely, miR-132 along with miR-212 was found to be upregulated in cardiomyocytes following hypertrophic stimuli [56]. miR-212/132 null mice were protected from pressure-overload-induced HF, whereas cardiomyocyte-specific overexpression of the miR-212/132 family promoted pathological cardiac hypertrophy, HF and death in mice. This effect is attributed to their direct effect on anti-hypertrophic and pro-autophagic FoxO3 transcription factor which hyperactivates the pro-hypertrophic calcineurin/NFAT signaling and thereby impaired the autophagic response upon starvation. These controversial reports could be justified by the different targets of miR-132 at different time-points of the disease. Overexpression of miR-132 due to hypertrophic stimuli suggests its role as a mediator to initiate cardiac gene reprogramming that can promote angiogenesis after ischaemic insult. However, experimental and clinical studies at a larger extent are required to support these speculations.

### Other miRs in DHD

In addition to the cardiovascular miRs reviewed above, a number of other miRs, not restricted to cardiovascular system have also been implicated in the pathophysiology of DHD. Although reflecting a more ubiquitous expression profile, recent studies have linked their misexpressed signatures with the etiology of DHD. Several groups have implemented microarray technology to analyse the expression pattern of miRs in murine and/or human diabetic and non-diabetic heart [113,121-123]. Shen et al. [121] provided strong evidence of activation of MAPK signaling pathway at hypertrophic stage in diabetic mouse heart and in cardiomyocytes exposed to HG. The important finding was that p38, a member of MAPK pathway was found to regulate the expression of miR-373 in glucose-induced cardiomyocyte hypertrophy. Further, hypertrophic cardiomyocytes transfected with miR-373 mimic showed repression of MEF2C, a transcription factor known to be involved in cardiac hypertrophic growth [83]. Taken together, the study revealed that miR-373 is transcriptionally regulated by p38 MAPK and miR-373 can protect against glucose-induced hypertrophy by repressing the hypertrophic protein, MEF2C.

A recent study determined the levels of miRs using Insulin2 mutant (Ins2+/−) Akita mice model of DCM. Among the other described miRNAs, miR-223, an anti-inflammatory and cardioprotective miR was also found to be downregulated. The increased level of pro-inflammatory TNFα and attenuation of anti-inflammatory mediator, IL-10 in Akita hearts were in line with the downregulation of miR-223 [113]. However, another study points to a different role of miR-223 in diabetic heart [122]. Quantitative miR expression analyses of left ventricular biopsies from the type 2 diabetic patients with left ventricular dysfunction revealed the upregulation of miR-223. Adenoviral-mediated overexpression of miR-223 in neonatal rat ventricular myocyte (NRVM) was found to increase the cellular glucose uptake, an effect which was independent of phosphoinositide 3-kinase (PI3K) signaling or AMP Kinase activity. Indeed, miR-223 overexpression-induced Glut4 protein expression in cardiomyocytes was required for increased glucose uptake. Furthermore, miR-223 inhibition in vivo using mmu-miR-223 inhibitor administered to ten week old C57BL/6 male mice at a dose of 80 mg/kg resulted in significant decrease in Glut4 expression. These gain and loss-of-function experiments demonstrated the post-transcriptional regulation of Glut4 by miR-223. The upregulation of miR-223 in the insulin resistant human heart can be explained as the adaptive response of miR-223 to restore the Glut4 expression and normalize the glucose metabolism in diabetic heart. These observations are also in concordance with the idea that miRs act as stress responsive elements and are responsible for modulating the cellular phenotypes under different pathophysiological conditions [124].

Elegant work by Greco et al. [123] identified the dysregulation of 6 miRs (miR-34b, miR-34c, miR-199b, miR-210, miR-223 and miR-650) in left ventricle biopsies obtained from diabetic patients with heart failure (D-HF) compared to the non-diabetic patients with heart failure (ND-HF), indicating specific pathogenetic mechanisms differentiating D-HF from ND-HF. The hypoxia-inducible factor (HIF) signaling pathway was found to be activated in D-HF patients, determined by the increased levels of HIF 1α mRNA in D-HF biopsies when compared to ND-HF. Of note, miR-210 is the downstream target of HIF 1α, a transcription factor that response to reduced O_2_ level and thereby activates an alternative cellular mechanism of ATP synthesis via upregulation of gene expression. Blumensatt and colleagues [125] demonstrated that an elevated secretion of activin A, an epicardial adipokine released by the epicardial adipose tissue (EAT) contributed to the inhibition of the Akt/GLUT4 pathway through upregulation of miR-143 in adult rat cardiomyocytes (ARC) that were exposed to the conditioned media (CM) generated from EAT of the patients with type 2 diabetes. Insulin-dependent Akt/Glut4 signaling pathway mediates cardiac glucose uptake by promoting the translocation of the glucose transporter GLUT4 to the plasma membrane. These findings revealed that upregulation of miR-143 in EAT by Activin A cause post-transcriptional gene silencing of oxysterol-binding protein-related protein 8 (ORP8) that resulted in inhibition of the insulin-mediated phosphorylation of Akt/eNOS-signaling pathway. The authors extended these findings to another study using human vascular smooth muscle cells, wherein the upregulation of miR-143 was also found to impair the insulin signaling via downregulation of ORP8 [126].

miR-301a was found to directly modulate the expression of potassium channel Kv4.2 at post-transcriptional level in right and left ventricles of diabetic (db/db) mice [127]. Using loss-of-function and gain-of-function approach in rat cardiomyoblasts (H9C2 cells) as an in vitro model, it was demonstrated that miR-301a directly regulate Kv4.2 by binding to Kv4.2 3’-UTR region. The observations in the in vitro model were consistent with the findings in the db/db mice ventricles where a significant increase in the expression of miR-301 and reduction in Kv4.2 expression showed a depleted repolarization reserve in the diabetic hearts was observed, suggesting the central regulatory potential of miR-301a in electrical cardiac conduction in diabetes.

miRs have also been linked to diabetes-mediated mitochondrial dysfunction which plays a credential role in the consequence of DCM. In a recently published article [128], miR-141 has been found to regulate the expression of IMM (inner mitochondrial membrane) phosphate transporter solute carrier family 25 member 3 protein (Slc25a3) in type I diabetic mice, treated with multiple low-dose STZ for 5 weeks. Slc25a3 is a transmembrane protein which is essential for ATP production as it provides inorganic phosphate from cytoplasm to the mitochondrial matrix [129,130]. Another protein, NADPH oxidase 4 (NOX4) has also been reported to be deregulated in diabetic heart [131]. Several recent studies demonstrated the upregulation of NOX4 expression under oxidative stress induced by hyperglycemia and hypercholesterolemia [132-134]. miR-25 has been recently evolved as a post-transcriptional gene silencer of NOX4 by directly targeting its 3′ UTR region. Initially, it was implicated in the regulation of insulin biosynthesis and NOX4 expression in diabetic nephropathy [132,135]. However, recently, miR-25 has been found to repress NOX4 expression in hypercholesterolemia-induced myocardial dysfunction rats [133]. In this study, authors showed a significant downregulation of miR-25 with a parallel upregulation of NOX4. Further, transfection of a miR-25 mimic into primary cardiomyocytes decreased oxidative stress, while a miR-25 inhibitor resulted in an upregulation of NOX4 protein and an increase in oxidative stress, suggesting the cardioprotective effect of miR-25 against oxidative stress. Although the role of miR-25 in diabetic myocardium still remains to be elucidated, since diabetes is a major inducer of oxidative stress, it is plausible that miR-25 would have similar functions in diabetic heart. Like miR-1, miR-25 has also been reported to repress the postnatal expression of Hand2 in cultured cardiomyocytes and in mouse hearts in vivo. In contrast, activation of calcineurin/Nfat signaling pathway under cardiac stress, resulted in Nfat-mediated transcriptional activation of the Hand2 gene and Nfat-mediated transcriptional repression of miR-25, which reawaken the expression of Hand2 genes, cardiac remodeling and HF [136].

Although the pathophysiological role of miRs remains fragmental, several studies discussed herein prompt the potential role of miRs in the pathophysiology of DHD. Given that miRs regulate cardiac gene expression and are released in to the circulation in heart disease, it may be speculated that they can act as both mediators and diagnostic biomarkers in DHD. Furthermore, understanding their release mechanism under pathological conditions as well as their regulatory role in cardiac gene expression may shed light on the mechanistic properties of miRs in DHD.

### Regulation of miRs expression and release in circulation under disease state

**Figure 3:**
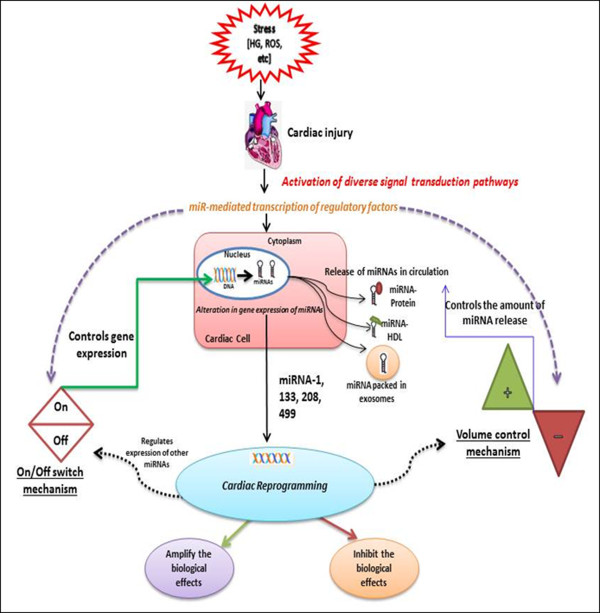
Proposed mechanism of modulated expression and release of cardiovascular miRs under pathophysiological conditions.

Although the research to clarify the expression and release of tissue-specific miRs into circulation is still in its infancy, it may be speculated that there are two major mechanisms that play important roles in regulating the expression and release of miR: A) “On/off switch mechanism” to control which gene is to be expressed. B) “Volume control mechanism” that increases or decreases the level of miR to be released in to the circulation (Figure 3). However, these mechanisms may be modulated under pathophysiological conditions like stress, HG, etc., due to activation of diverse signal transduction pathways [42,137]. Activation of signal transducers modulates the miRs that are key regulators of cardiac transcription [138]. Since miRs are known to regulate the gene transcription pathways by acting on specific mRNAs targets, they could modulate the transcription of regulatory proteins that further regulate the expression and release of specific miRs. Consequently, there is altered expression of genes encoding proteins that regulate the expression of miRs. A recent study [139] suggests that cardiac expressed miRs regulate the expression of other cardiac miRs due to their transcriptional modulation potential. Furthermore, cardiac miRs have been reported to initiate direct cardiac reprogramming to compensate the structural and functional loss to cardiac tissue after injury [140].

Since, diabetic heart disease is associated with altered cardiac gene expression, it may be reasonable to hypothesize that this miR-mediated cardiac reprogramming via On/Off switch mechanism and Volume control mechanism may trigger the transcription of specific proteins that are documented to regulate the expression of their specific miRs (as discussed in Table 1) via On/Off switch mechanism.

### Role of miRs in cardiac regeneration

Cardiac injury in diabetes is associated with inadequate regeneration, accumulation of excessive fibroblast and subsequent genetic alterations which impair the normal structure and function of the heart. An approach that can repopulate the injured heart with new and functional cardiomyocytes could be an ideal way to treat cardiac injury. Cardiac gene reprogramming could have the potential for regeneration of cardiomyocytes. miRs have been demonstrated to play crucial role in cardiac muscle development and differentiation (Table 1).

Recently, miR-92a and miR-210 are reported to play an important role in myocardial neovascularization, that may help in vascular repair after MI [141]. In addition, transplantation of mesenchymal stem cells (MSCs) that were overexpressed with miR-126 was shown to enhance angiogenesis in the infarcted myocardium of mice via AKT/ERK-related pathway [142]. Intriguingly, exogenous administration of hsa-miR-590 and hsa-miR-199a to neonatal and adult rat cardiomyocyte markedly stimulated proliferation and promoted cardiac repair. In vivo injection of the synthetic form of these miRs into the left ventricle of neonatal rats produced similar increase in proliferated cells even in the differentiated cells, suggesting the cardiac regenerative potential of hsa-miR-590 and hsa-miR-199a. Moreover, these miRs were able to boost the normally ineffective myocardial repair that occurred after MI [143]. Besides its role in cardiac pathophysiology, cardiac-specific miR-499 has been shown to regulate the differentiation and proliferation of human cardiomyocyte progenitor cells by repressing histone deacetylase 4 and Sox6 proteins [51]. miR-499 mediated cardiac differentiation has also been reported in human embryonic stem cells [144] and bone marrow mesenchymal stem cells [145]. Intriguingly, the combination of miR-1, miR-133, miR-208 and miR-499 in the presence of JAK inhibitor I were found to exhibit reprogramming potential by converting cardiac fibroblasts to cardiomyocytes (Figure 3) [140,146]. As stated above DHD remains asymptomatic for long time and when diagnosed at the late stage, a significant number of functional cardiomyocytes are replaced by fibroblasts. Therefore, reprogramming of fibroblasts to functional cardiovascular cells using miRs offers an exciting opportunity for cardiac regenerative therapy in people with diabetes.

### Can miRs be used as a clinical diagnostic tool for DHD?

Recent studies have clearly shown that heart disease in diabetes develops at a much earlier stage before it is clinically diagnosed and therefore, timely management may halt the progression of the disease [12,76,77]. Diastolic dysfunction in diabetes occurs as a consequence of molecular changes at gene level. These changes are attributed to the time-dependent change in cardiac gene expression [23,147]. Therefore, an attempt to diagnose the disease at molecular level can be a valuable strategy towards effective therapeutic management in patients with DHD. However, this requires the development of effective diagnostic tool for the early diagnosis of the disease. Currently, there are no specific and reliable tools to detect the early stages of the disease. The available clinical diagnostic tools such as echocardiography, coronary angiography and levels of circulating biomarkers can only diagnose established disease [148]. Recently developed non-invasive multi-slice computed tomography angiography can be used to detect heart disease in asymptomatic type 2 diabetic patients, however, it has disadvantages of high radiation exposure and costs [63,149]. Echocardiography has been considered as a choice of non-invasive test to examine the structural and functional alterations in heart diseases. However, this method has few limitations that restrict its use as a reliable tool for the diagnosis of heart disease. Severe diastolic dysfunction is often not identified in patients with pseudonormal transmitral flow due to high left atrial pressure, thus limiting the use of echocardiography for accurate diagnosis of myocardial dysfunction in such group of patients [150]. Moreover, detection of systolic dysfunction has been found to be more challenging in human studies using echocardiography due to insensitivity of standard parameter of systolic function, ejection fraction (EF) [151]. In addition these advance methodologies requires the patient to visit the specialty centre.

Conventional circulating biomarkers such as aspartate aminotransferase, total lactate dehydrogenase, CK and CK-MB are no longer recommended in clinical diagnosis of cardiac injury due to their low sensitivity, less precision, wide distribution in many tissues and relatively limited specificity for the disease [152]. Cardiac troponins (cTn) have been widely used ‘gold standard’ biomarkers in clinical assessment of myocardial injury. The major limitations with conventional cardiac troponin is its poor sensitivity and doesn’t change unless there is substantial damage in the heart such as myocardial infarction [153], presenting low serum concentrations in the early hours after the onset of chest pain [154]. Recently developed High-sensitivity cardiac troponin assays (Hs-cTn) and copeptin have shown a higher diagnostic accuracy as compared to conventional cTn [155,156]. However, these biomarkers can be used only in the established disease settings [154,156]. In a clinical study [150] carried out on type 2 diabetic patients, the diagnostic potential of circulating brain natriuretic peptide (BNP) on systolic and diastolic function was examined. It was found that BNP and echocardiography were not sufficiently sensitive to identify the subclinical dysfunction, thereby augmenting the need for more sensitive biomarkers. Another major limitation of these biomarkers is their specificity to the disease, especially in elderly patients with acute myocardial infarction (AMI) [152]. Accurate diagnosis of AMI in geriatric population is a daunting challenge due to various co-morbidities, such as end-stage renal disease, chest discomfort and only slight elevation in circulating troponin levels [157]. In addition, non-specific elevation of troponin levels in non-ischemic conditions such as thrombotic acute coronary syndrome (ACS), congestive heart failure (CHF) as well as their low serum levels in elderly patients with renal failure further challenge its diagnosis potential for AMI [158]. Elderly patients comprise about one-half of the diabetic population [159,160] and AMI impose a major complication among diabetics. So the overall reliability on diagnosis performance of these biomarkers in diabetic population is unknown [161]. A clinical study by Ambhore et al. [162] demonstrated that non-invasive techniques like electrocardiogram (ECG) failed to diagnose the nonischaemic cardiomyopathy in diabetic subject with normal coronary artery, indicating the importance of an accurate and reliable diagnostic tool for diabetic patients. Collectively, these shortcomings augment the discovery of highly specific and sensitive biomarkers for the detection of DHD. In this respect, circulating cardiovascular miRs have gained considerable interest as potential diagnostic biomarkers for the detection of cardiovascular diseases. An overview of clinical studies suggesting the diagnostic potential of miRs in diabetic patients is shown in Table 2.

**Table 2:** Overview of clinical studies showing differentially expressed circulating miRs in diabetic patients

**S.No**	**Type of patients**	**Study cohort**	**Source of miR isolation**	**Most differentially expressed miRs**	**Method of analysis**	**Major findings**	**References**
1	T2DM	822 individuals from Bruneck study	Plasma	Downregulation of miR-20b, -21, -24, -15a, -126, -191, -197, -223, -320, -486; Upregulation of miR-28-3p	MiR microarray profiling; qPCR	First study to identify differential miR signature in T2DM patients.	[31]
						Plasma levels of some miRs changed before the manifestation of diabetes, suggesting miRs as early predictive tool in diabetes and vascular disease	
2	T2DM	56 subjects: 18 cases of newly diagnosed T2DM patients (n-T2DM); 19 cases of pre-diabetics and 19 cases of T2DM-susceptible individuals with normal glucose tolerance (s-NGT)	Serum	Upregulation of miR-9, -29a, -30d, -34a, -124a, -146a and −375 in n-T2DM.	qPCR	Significant change in expression of diabetes-related miRs in n-T2DM whiles no dramatic change in pre-diabetics and s-NGT.	[163]
						miR-34a most significantly changed in all the 3 study groups.	
3	T2DM and obese	13 patients with T2DM; 16 obese patients (OB) with T2DM; 20 obese patients (OB-T2DM) and 20 healthy volunteers.	Serum	miR-15b, -138, -376a and −503	Pre-screening with pre-aliquoted miR PCR panels and validation of selected miRs by qPCR	First study to identify differential miR signature in T2DM patients, OB, OB-T2DM and healthy subjects.	[164]
						Biomarker potential of miR-15b, miR-138 and miR-376a to differentiate OB from OB-T2DM and T2DM.	
						Biomarker potential of miR-138 and miR-503 to differentiate T2DM from OB-T2DM.	
4	T2DM, Individuals with or without metabolic syndrome	265 individuals: n = 50 with metabolic syndrome; n = 50 with T2DM; n = 89 with hypercholesterolemia; n = 30 with hypertension and n = 46 healthy controls	Blood	miR-23a, -27a, -103, --132, -150, -192, -195, -197, -320a, -375, and −509-5p	MiR microarray profiling; qPCR of selected miRs	Correlation between aberrant miR expression and risk factors in diabetes and its vascular complications.	[165]
5	T2DM with or without vascular complications	36 individuals: 12 T2DM patients without any chronic complications; 12 T2DM patients with macrovascular and 12 T2DM patients with microvascular complications.	Serum	52 miRs in T2DM with macrovascular and 68 miRs in T2DM with microvascular complications	MiR microarray profiling; qPCR of selected miRs	Upregulation of miR-31a in T2Dm with microvascular complications	[166]
6	T1DM	Hvidoere (275 T1DM patients), Danish (129 T1DM patients) and Copenhagen Puberty (151 T1DM patients) Cohorts	Serum	Upregulation of miR-24, -25, 26a, -27a/b, -29a, -30a-5p, -148a, -152, -181, -200a and −210	MiR microarray profiling; qPCR	miR-25 negatively associated with beta-cell function and positively associated with glycaemic control	[167]

The discovery of miR as regulators of molecular targets involved in pathogenesis of cardiovascular diseases has uncovered a new and exciting way for the diagnosis of DHD. However, our knowledge on which miRs are altered in DHD is largely incomplete. Tissue-specific miRs have recently been detected in circulation and exhibit unique expression pattern in disease states. Only a few years ago, miRs were detected in serum and plasma in a remarkably stable form even under different conditions, such as, boiling, high/low pH, incubation for long time at room temperature and multiple freeze-thawing cycles [168,169]. Due to their stability in different human fluids and the ease by which they can be detected quantitatively, they have been highlighted to be used as potential diagnostic biomarkers for cardiovascular diseases such as myocardial infarction, coronary artery disease, and HF [71,170].

Sudden induction of myocardial cell death is reported to induce the immediate release of cardiovascular miRs into circulation where they can be detected at the initial stage of AMI [171,172]. Several groups have studied the diagnostic performance of circulating cardiovascular miRs in heart diseases although their role in DHD is yet to be explored. A consistent elevation in plasma levels of four cardiac miRs (miR-1, -133, -208a and −499) was observed in AMI patients within few hours after the onset of symptoms for infarction [172-181]. Wang et al. [171] documented the elevated plasma levels of these cardiac specific miRs in rat models of AMI as well as in AMI patients. While the level of miR-1, -133 and −499 were expressed at very low level, miR-208 was not at all detected in the plasma of healthy individuals. Interestingly, miR-208a was readily detectible in 91% patients with AMI within 4 h of sample collection while parallel cTnI was detected only in 85% of patients. This study suggests the superiority of miRs in diagnosing AMI at an early stage when compared to troponin. Long et al. [182] compared the plasma levels of miR-1 and −126 against cardiac troponin I (cTnl) in patients with AMI. The expression pattern of miR-1 and −126 were found to be similar to cTnI levels. Strikingly, in another study the plasma peak concentration of miR-1 and −133 was found to be significantly higher when compared to cTnI after 2 h of the onset of chest pain [174]. These evidences suggest that the circulating cardiovascular miRs could be sensitive and more specific markers for the diagnosis of AMI.

Geriatric patients with non-ST segment elevation myocardial infarction (NSTEMI) present atypical symptoms and inconclusive ECG due to various co-morbidities such as left bundle-branch block without chest pain [183], ECG changes due to the presence of cardiac pacemaker [183] as well as due to coexistence of chronic ischemic cardiomyopathy [184]. As a consequence, aged population have higher incidence of unrecognized AMI. Recently, miRs are also reported to exhibit greater sensitivity and specificity than conventional cTn and Hs-cTn biomarkers for geriatric population where cTn levels are low and not reliable for detection and prognosis of NSTEMI in elderly population [158,185]. Supporting this, a recent study by Olivieri et al. [186] investigated 3–10 fold increase in plasma levels of miR-1, miR-21, miR-133a and miR-423-5p along with >80-fold elevation of miR-499-5p level in NSTEMI patients as compared to control subjects. Interestingly, the expression of miR-499-5p and miR-21 was significantly increased in NSTEMI patients as compared to CHF patients. These miRs were able to differentiate between NSTEMI elderly patients, CHF patients as well as control individuals where their diagnostic accuracy was found to higher than cTn, suggesting their greater sensitivity and specificity for diagnosis of AMI as compared to cTn.

**Figure 4:**
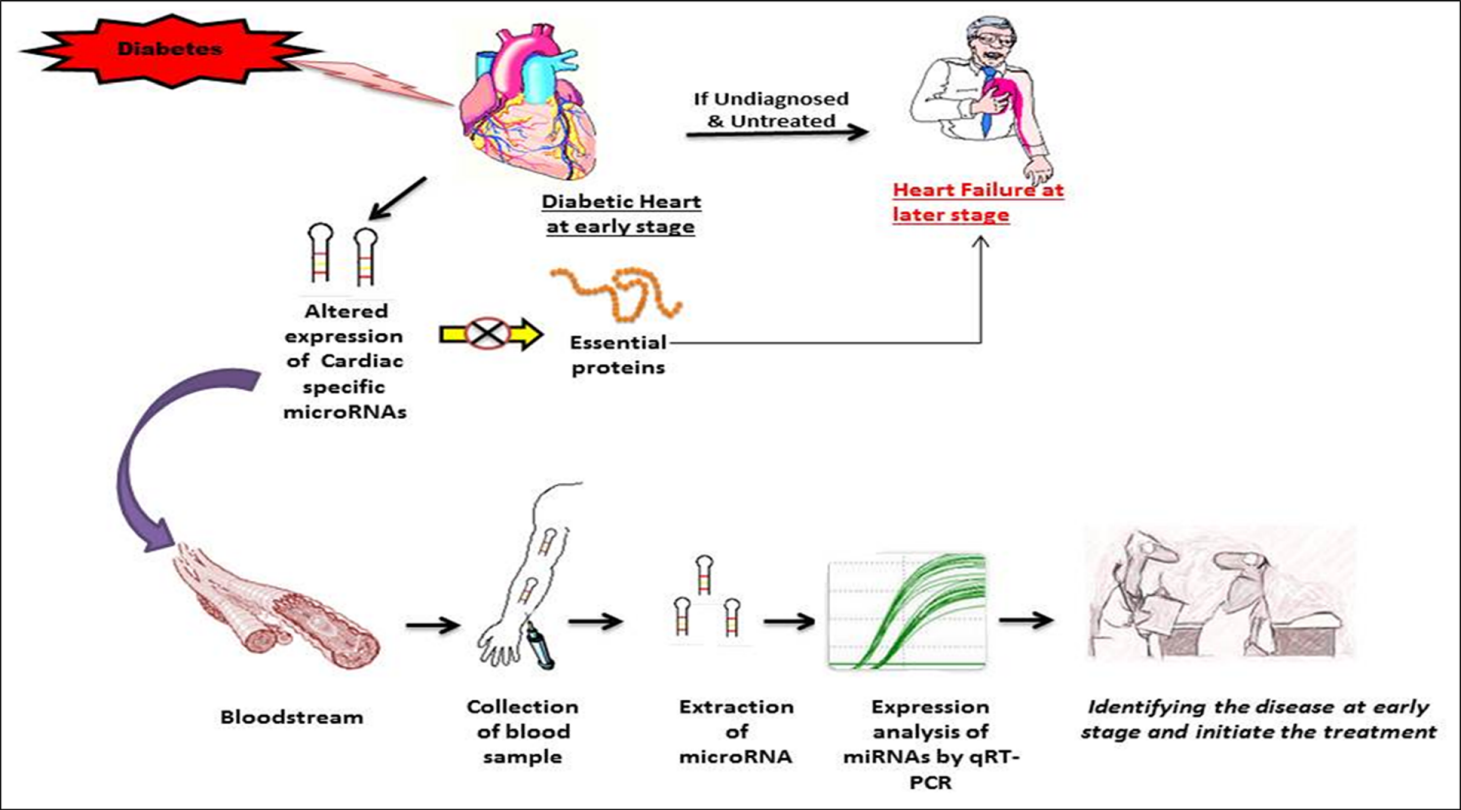
**Supporting the hypothesis that miRs could be a potential diagnostic biomarker for early diagnosis of diabetic heart disease.** Heart disease in diabetes develops at a much earlier stage before it is clinically diagnosed and if undiagnosed and untreated, it may lead to HF at later stage. Diabetes is associated with marked molecular changes in the heart produced by miRs. These molecular changes occur before any structural and functional alteration in diabetic heart. Altered expression of miRs in diabetic heart inhibits the synthesis of essential proteins required for the normal cardiac physiology. Parallel to this, these cardiovascular miRs are also released into the bloodstream, where they can be easily detected as biomarkers for DHD.

Vascular complications in diabetes start with impaired angiogenesis and endothelial dysfunction which lead to atherosclerosis at later stage. Endothelium-expressed miRs: miR-126 and miR-132 are recently documented to be altered significantly in diabetes and HF [31,55,56,118,119], which suggests their potential to be used in diabetes associated coronary artery disease. A large prospective population-based study by Zampetaki et al. [31] was the first study to determine the changes in plasma miR signature in T2DM patients. Global miR profiling of plasma samples obtained from the study cohort (n = 822) identified a group of differentially expressed plasma miRs in diabetic subjects when compared with age and sex matched non-diabetic subjects. Of 13 plasma miRs identified to be differentially expressed, the most significant difference was observed in the expression of miR-20b, miR-21, miR-24, miR-15a, miR-126, miR-191, miR-197, miR-223, miR-320, and miR-486, which were found to be less abundant in diabetic patients. However, the expression of miR-28-3p was found to be moderately increased. In addition, the differences in miR levels were also replicated in plasma samples obtained from hyperglycaemic Lep^Ob^ mice, a type 2 diabetes mice model. Importantly, the expression profile of 5 plasma miRs (miR-15a, miR28-3p, miR-126, miR-223, and miR-320) formed a unique signature pattern sufficient to correctly distinguish between individuals with prevalent and incident diabetes from healthy controls. Of note, plasma levels of miR-15a, miR-126, and miR-223 were significantly reduced in diabetic subjects compared to non-diabetics before the manifestation of diabetes, providing evidence that the determination of the level of these small RNA elements in the blood samples might serve as early predictors in diabetes and its vascular complications. In addition, miR-126 content was also reduced in endothelial apoptotic bodies in the entire cohort in a glucose-dependent fashion, concurring to its potential role as diagnostic biomarker for risk estimation of vascular complications in diabetic individuals. Fichtlscherer et al. [119] studied the relation of circulating levels of vessel wall and inflammatory cell-derived miRs in CAD patients, thereby exploring their potential as diagnostic biomarkers for patients with CAD. They showed profound downregulation of vascular miRs, including miR-126, 17 ~ 92 cluster (miR-17, miR-20a, miR-92a), miR-130a, miR-221, members of the let-7 family (let-7d), and miR-21. In contrast, cardiac-specific miRs, such as miR-133 and miR-208a were significantly upregulated in CAD patients. In their prospective study, the authors initially performed a high-throughput microarray to profile the changes in circulating miRs level in a small number of CAD patients and healthy volunteers (n = 8 in each group). 46 miRs were detected to be significantly downregulated while 20 miRs were significantly upregulated. These observations led to further analyses of selected miRs in two distinct larger study cohorts: derivation cohort (n = 36 CAD patients versus n = 17 healthy volunteers) and validation cohort (n = 31 CAD patients versus n = 14 healthy volunteers). Interestingly, most of the differentially expressed circulating miRs detected in CAD patients of both cohorts are known to be expressed in the vasculature (particularly in endothelial cells) and in cardiac and/or skeletal muscle cells. The reduced circulating levels of endothelial miRs (miR-126) in disease patients in both the studies was striking, because miR-126 is expected to be increased in cardiovascular disease as a compensatory mechanism to induce vasculogenesis. Indeed, the reduced levels of circulating vascular miRs in CAD patients may be caused by increased uptake of these miRs into atherosclerotic lesions. This could also be the case in diabetic subjects with undiagnosed endothelial dysfunction. These pioneering studies might therefore, open the door to future investigations aiming to delineate the intricate nature of miRs in various cardiovascular diseases including DHD. A pictorial representation of the use of cardiovascular miRs as diagnostic biomarkers is depicted in Figure 4.

### Future directions and conclusion

Since their discovery, cardiovascular miRs have emerged as the key players in regulating cardiac gene expression under normal and disease states. Given that miRs coordinately regulate the complex webs of cardiac gene networks and that DHD is associated with altered gene expression, it is tempting to speculate that these ‘micro’ elements may control ‘macro’ phenotypes in diabetic heart. Although our understanding of cardiovascular miR biology in diabetic heart is still in its infancy, the literature reviewed herein suggests that these miRs may have crucial role in the pathophysiology of DHD and therapeutic manipulation of these miRs can be a useful strategy towards the management of the disease. Furthermore, modulated levels of these circulating cardiovascular miRs before the clinical manifestation of disease augment the opportunities to provide new clues for early detection of heart disease in diabetic subjects, for disease prognosis and for assessing the efficacy of therapeutic interventions. However, the vision of miRs as successive therapeutic strategy and its clinical relevance in DHD is a bit hazy due to limited knowledge about their role in DHD. As discussed in this article, the use of different models studied, cell-types, disease states as well as the lack of large size population and differences in key parameters such as age, sex, and pharmacological treatments in clinical studies could affect and contribute to the complex phenotypes of these miRs. Furthermore, developing miR-based therapies would be a big challenge and several hurdles need to be crossed before considering them for clinical treatments. Most importantly, due of their pleiotropic profile, miRs can target several genes, that could bring about undesired ‘off-target’ side effects. Future studies aimed at understanding the multiple pathways regulated by cardiovascular miRs at different stages of disease employing advanced bioinformatics technologies are required to develop them as innovative therapeutic targets. In addition, studies based on larger size populations and using standardized protocols for controlling multiple factors that could affect the miR expression are requisite to elucidate the scope of using cardiovascular miRs as potential diagnostic biomarkers for DHD. Nevertheless, additional understanding into the regulatory mechanisms and release profile of cardiovascular miRs in diabetes would help us to develop effective therapeutic interventions along with early detection of DHD.

## Competing interests

The authors declare that they have no competing interests.

## Authors’ contributions

SR performed extensive literature search and wrote the first draft of the manuscript: PM provided details on clinical studies and did extensive revision of the manuscript; RK designed the manuscript, performed literature search and drafted the manuscript. All authors read and approved the final manuscript.
